# Low social acceptance among peers increases the risk of persistent musculoskeletal pain in adolescents. Prospective data from the Fit Futures Study

**DOI:** 10.1186/s12891-022-04995-6

**Published:** 2022-01-13

**Authors:** Henriette Jahre, Margreth Grotle, Kaja Smedbråten, Kåre Rønn Richardsen, Pierre Côté, Ólöf Anna Steingrímsdóttir, Christopher Nielsen, Kjersti Storheim, Milada Småstuen, Synne Øien Stensland, Britt Elin Øiestad

**Affiliations:** 1 Department of Physiotherapy, Faculty of Health Sciences, OsloMet – Oslo Metropolitan University, Postboks 4 St. Olavs plass, 0130 Oslo, Norway; 2 Research and communication unit for musculoskeletal health (FORMI), Division of Clinical Neuroscience, Oslo University Hospital, Oslo, Norway; 3 Faculty of Health Sciences, Ontario Tech University, Oshawa, Canada; 4 Division of Mental and Physical Health, Norwegian Institute of Public Health, Oslo, Norway; 5 Department of Pain Management and Research, Oslo University Hospital, Oslo, Norway; 6 Norwegian Centre for Violence and Traumatic Stress Studies (NKVTS), Oslo, Norway

**Keywords:** Musculoskeletal pain, Social acceptance, Adolescents, Risk factor, Psychological distress

## Abstract

**Background:**

Musculoskeletal pain has a high prevalence in adolescence and causes huge consequences for the individuals and the society. Little knowledge exists on social risk factors for musculoskeletal pain in adolescents. This study aimed to investigate if low social acceptance among peers during the first year of upper secondary school was associated with persistent and severe persistent musculoskeletal pain 2 years later and if psychological distress modified this association.

**Methods:**

Longitudinal data from the Norwegian Fit Futures Study was used. Students in the first year of upper secondary school answered an electronic questionnaire, covering health status, pain, social acceptance among peers, and psychological distress. Persistent musculoskeletal pain was measured 2 years later. Multiple logistic regression analyses and moderation analyses were conducted adjusting for sex and chronic diseases. Main analyses were conducted on participants without persistent musculoskeletal pain at baseline, and secondary analyses were conducted on all participants with and without persistent musculoskeletal pain at baseline.

**Results:**

Of 775 participants (52% females), 556 (71.7%) were pain-free at baseline and included in the main analyses. Significant associations between low social acceptance among peers and persistent musculoskeletal pain 2 years later were found in crude (Odds ratio (OR) = 1.8, 95%CI [1.0–3.1]) and adjusted analyses (OR = 1.8, 95%CI [1.0–3.2]). No statistically significant effect modification of psychological distress (*p* = 0.89) on this association was found. A significant association between low social acceptance and persistent musculoskeletal pain was found in adjusted secondary analyses of all the students (*n* = 692) (OR = 1.6, 95%CI [1.0–2.3]).

**Conclusions:**

Our results indicate that low social acceptance among peers increases the risk of future persistent musculoskeletal pain in adolescents. Thus, interventions strengthening adolescent’s social arenas may be helpful to prevent persistent musculoskeletal pain.

**Trial registration:**

Retrospective registered at clinicaltrials.org NCT04526522.

**Supplementary Information:**

The online version contains supplementary material available at 10.1186/s12891-022-04995-6.

## Background

Musculoskeletal (MSK) pain is common and ranked by the Global burden of disease study as the number one cause of years lived with disability (YLD) among all health conditions [1]. The prevalence of persistent MSK pain is high already during adolescence [2, 3]. Adolescents experiencing persistent MSK pain have a high risk of developing long-lasting MSK pain and show high use of health care services in adulthood [4, 5]. Identifying modifiable risk factors are necessary to develop effective interventions aiming to prevent future persistent MSK pain.

Several studies have investigated risk factors for persistent MSK pain in adolescents [6, 7]. However, as highlighted in a recent systematic review, the quality of the evidence is, in general, low [6]. Furthermore, while many existing studies have investigated anthropometric factors (sex, height, body mass index (BMI)) and lifestyle factors, such as physical activity, little attention has been given to social factors [6, 7].

One important social factor in adolescence is perceived peer acceptance, often referred to as the experience of being socially accepted and being liked among peers [8]. Life-course epidemiology suggests that adolescence is a vulnerable period of life when peer relations are of particular importance, and the feeling of being socially accepted and liked among peers is critical in developing life-long social, emotional, and behavioural skills [9]. Social relationships promote adaptive behaviour to health stressors and may act as a “buffer” against pain and poor health [10]. Negative aspects of social relationships such as bullying at school [11], loneliness [12], and peer-related stress [13] have been reported to increase the risk of MSK pain in adolescence.

Another important health outcome with high prevalence among adolescents is psychological distress, including symptoms of anxiety and depression [14]. Research has shown that psychological distress during adolescence may impact adolescent’s social life in terms of social exclusion and loneliness [15] and to increase the long term risk of MSK pain [6]. Different levels of psychological distress might influence the longitudinal relationship between social acceptance among peers and future MSK pain differently and therefore act as a potential effect modifier of the association. Investigating these associations in a longitudinal study is helpful to better clarify this relationship. To our knowledge, no previous studies have investigated the relationship of low social acceptance among peers and persistent MSK pain with psychological distress as a potential effect modifier.

The objectives of this study were to investigate whether: i) low social acceptance in the first year of upper secondary school was associated with later onset of persistent MSK pain and severe persistent MSK pain within 2 years, and ii) whether psychological distress was an effect modifier of these associations.

## Methods

### Study population

**Fig. 1:**
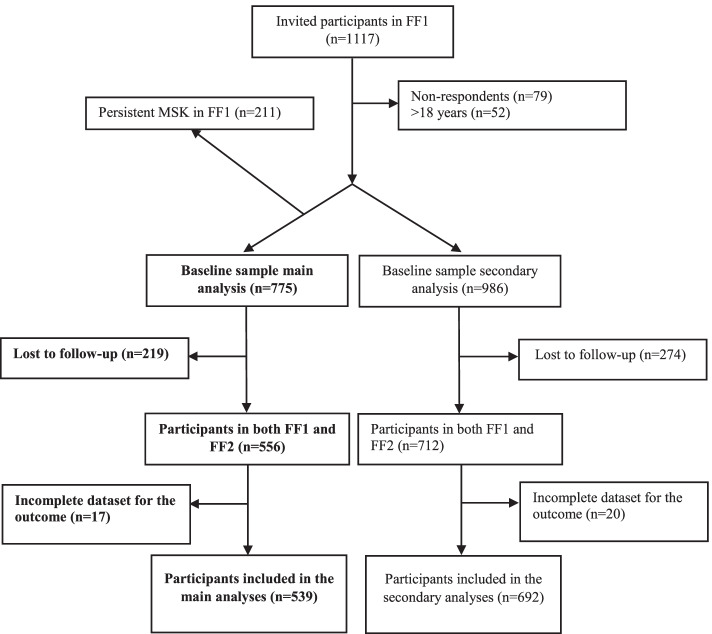
Flow-chart of study participants. Main analysis = without pain at baseline, secondary analysis = all study participants

This prospective cohort study used data from the Fit Futures (FF) study. The Fit Futures study is a population-based cohort study of adolescents aiming at following adolescents’ lifestyle and health status over time. In the first wave of the study (FF1), conducted 2010–2011, all first-year upper-secondary students in the municipalities of Tromsø (urban) and Balsfjord (rural) in Northern Norway were invited (*N* = 1117) and *N* = 1038 participated, yielding a response rate of 93%. In the second wave (FF2), conducted in 2012–2013, all third-year upper-secondary students in the same schools and all FF1 participants, irrespective of educational status and school district, were invited (*N* = 1130). *N* = 868 participated for a response rate of 77%. All participants completed online questionnaires, a clinical interview, anthropometric measurements and medical examinations at the research unit at the University Hospital of Northern Norway. For further details regarding the study, see [16, 17]. Our sample was restricted to adolescents who had participated in both study waves. We define the “population at-risk” of developing persistent MSK pain as adolescents without persistent MSK pain in FF1; therefore, we excluded those with persistent MSK pain at baseline (*n* = 211) from the main analysis. Adolescents older than 18 years in FF1 were excluded (*n* = 52). Three percent (*n* = 17) of the cohort had missing outcome data and were also excluded. This resulted in a sample of 539 participants in the main analyses. Secondary analyses were conducted on a mixed sample of both participants with and without persistent MSK pain at baseline (*n* = 692) (Fig. 1).

### Ethical considerations

Participation was voluntary and based on written informed consent. Written permission from a guardian was required for participants under the age of 16 years. The Regional Committee for Medical and Health Research Ethics in Norway (2019/599/REK Nord) and the Norwegian Centre for Research Data (954769) approved the current study. The study protocol for the present analysis has been published at clinicaltrails.gov (NCT04526522). Reporting of this study follows the Strengthening the Reporting of Observational Studies in Epidemiology (STROBE) statement (Additional file 1) [18].

### Outcome

The primary outcome was collected through the electronic questionnaire in Fit Futures and defined as *persistent MSK pain,* assessed with the following questions, “*Do you have persistent or recurrent pain that has lasted for three months or more?”* The responses were “yes” or “no.” If participants answered yes, they were asked, *“How often do you have pain?”* with four response alternatives: “*constantly, without a pause”*, “e*very day, but not all the time”*, “*every week, but not every day”*, and “*rarer than every week”*. Then, participants were asked, *“where does it hurt”,* with 14 body regions as response alternatives. This questionnaire was developed specifically for the Fit Futures study. Pain in the shoulders, arm/elbow, hand, hips, thigh/knee/shin, ankle, neck, upper back, and lower back were defined as MSK pain. We defined persistent MSK pain as pain experienced at least once per week over the last 3 months in at least one body site. MSK pain at baseline was assessed with the same questionnaire.

The secondary outcome was *severe persistent MSK pain*, assessed with the same questions as for the primary outcome, adding information about pain intensity rated on a numeric rating scale from 0 (no pain) to 10 (worst pain imaginable). Severe persistent MSK pain was defined as pain at least at one site once per week over the last 3 months with an intensity of at least 5/10 [19].

### Exposure variable

Social acceptance among peers was measured with five questions from the revised Norwegian version of Harter’s Self-perception Profile for Adolescents; scale for social competence [20, 21]. This subscale has proven good reliability and validity among Norwegian adolescents [22] and consists of five questions concerning the adolescent’s perception of ease of making friends and being socially accepted by peers. Participants were asked whether they *“find it hard to make friends”, “have many friends”, “are hard to like”, “feel popular among peers, and “feel accepted among peers”.* The responses were scored on a four-point scale ranging from “highly correct” (4 points) to “highly incorrect (1 point)”. The two negative worded items were reversed, and the average item score was calculated by dividing the total score by the number of items (range 1–4), as suggested by the developers [20]. A higher score indicated a higher level of perceived social acceptance among peers. Because there was little variation in social acceptance data, this variable was dichotomized according to normative values identified in a previous large-scale study of Norwegian adolescents with a cut-off for low social acceptance among peers of ≤3.0 [20].

### Potential effect modifier

Psychological distress, including symptoms of anxiety and depression, was measured by the Hopkins Symptoms Checklist-10 [23], which is validated in Norwegian adolescents [24]. The questionnaire consisted of 10 items measuring whether the adolescents had been bothered with the feelings: “*sudden fear for no reason”, “felt afraid or worried”, “felt faintness or dizziness”, “felt tense or upset”, “self-blame”, “sleeplessness”, “depression or sadness”, “felt useless or worthless”, “felt that life was a struggle”,* and/or *“the feeling of hopelessness”.* Each item was answered on a four-point scale ranging from “not at all” (1 point) to “extremely” (4 points). A mean score was calculated (range 1–4), as described by the developers [23], and a higher score indicates more symptoms of psychological distress. The score was dichotomized using a well-established cut-off (> 1.85 = symptoms of psychological distress) [24].

### Background variables and possible confounders

Age, objectively measured BMI, and persistent MSK pain were measured at baseline and used to describe the study sample. BMI was categorized into age-adjusted cut-offs from Cole and Lobstein as *“thinness”, “normal weight”*, and *“overweight/obese*” [25]. Information regarding sex, comorbidities, and parent’s education were collected and used as potential confounding factors based on theory and previous empirical findings [26, 27]. Sex was measured as girls/boys, and chronic diseases were measured with the question: *“Do you have any chronic or persistent diseases?”* categorized as yes or no. Parent’s education was a presumed confounder [6], but due to a large number (24–30%) of adolescents not knowing their parent’s education level, it was not included in the analyses.

### Statistical analyses

Descriptive data were presented as means and standard deviations (SDs) when continuous and categorical data were reported as counts and percentages. A two-year incidence rate of new cases with persistent MSK pain at follow-up was calculated. The two-year incidence was calculated by dividing the number of participants who developed a new episode of persistent MSK pain at follow-up by the number of participants at risk at baseline (study sample). Analyses were conducted to assess possible attrition bias by comparing baseline characteristics between participants lost to follow-up and respondents. Independent sample t-test was used to compare normally distributed pairs of continuous data, and categorical variables were compared using the chi-square test.

Univariate logistic regression was used to estimate the crude association between social acceptance and persistent MSK pain. Multiple logistic regression analyses were used to include sex and comorbidities as confounding factors in the model, based on previous studies [26, 27]. The results were presented with odds ratios (ORs) and 95% confidence intervals (Cis). Due to the low number of missing values (0.1–3.5%) on exposures and confounders, we only performed complete-case logistic regression analyses [28].

**Fig. 2:**
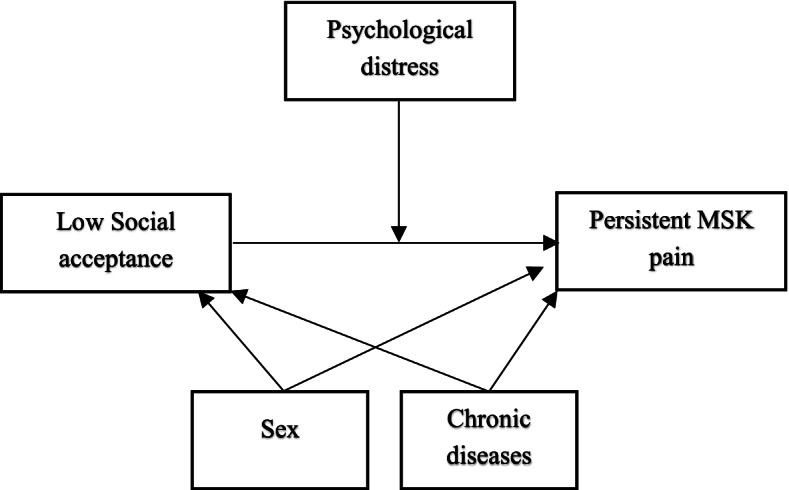
Conceptual diagram of the moderation model. MSK = musculoskeletal; Social acceptance measured by a subscale from Self-perception profile for adolescents. Low social acceptance ≤3. Psychological distress measured by Hopkins symptom check list-10 (1–4), psychological distress ≥1.85

To investigate if psychological distress was an effect modifier, a moderation analysis was conducted. In this model, social acceptance was included as the exposure, persistent MSK pain as outcome, and psychological distress as a possible moderator (Fig.2). The moderation analysis was performed according to Hayes using PROCESS macro in SPSS, model 1 [29]. A bias-corrected bootstrap method with 5000 bootstrap samples was used to estimate the effect modifier’s confidence intervals. In addition to the moderation analysis, univariate regression analyses investigating the association between social acceptance among peers and persistent MSK pain were conducted in a sample stratified into low and high level of psychological distress to observe potential differences in the magnitude or direction of the association in these different subsamples. Due to few cases of persistent MSK pain, multiple regression could not be fitted with sufficient precision. Moderation analysis was not possible to conduct for the secondary outcome due to too few cases of MSK pain.

To investigate the potential impact of incidence-prevalence bias on the measures of association, we performed secondary analyses of the whole cohort (*n* = 692), including all participants with and without persistent MSK pain at baseline. Associations with a significance level of ≤0.05 were considered statistically significant. All analyses were considered exploratory so no correction for multiple testing was done. All statistical analyses were conducted using SPSS statistical software version 27 (SPSS Inc., Chicago, IL, USA).

## Results

### Demographics of participants

The mean age at baseline was 16.1 (SD 0.5) years, and 52% of the sample were females (Table 1). The reported prevalence of chronic diseases was 25%. Fourteen percent of the study sample reported symptoms of psychological distress, and 28% reported having low social acceptance among peers (Table 1). Of the participants lost to follow-up (*n* = 219), 68% were males. More adolescents lost to follow-up did not know their parent’s educational level. No differences were found in social acceptance data, nor psychological distress in participants lost to follow-up than those who remained in the study (Additional file 2).

**Table 1:** Baseline characteristics of the study sample

Variables	Baseline sample ***n*** = 775	Follow-up participants, main analysis ***n*** = 539	Follow-up participants, secondary analysis ***n*** = 692
Sex, females	355 (45.8)	280 (51.9)	381 (55.1)
Age (mean, SD)	16.1 (0.5)	16.1 (0.5)	16.1 (0.5)
BMI			
Thinness	38 (4.9)	29 (5.4)	34 (4.9)
Normal weight	551 (71.1)	392 (72.2)	504 (71.8)
Overweight/obese	183 (23.6)	117 (21.7)	153 (22.1)
*Missing*	*3 (0.4)*	*1 (0.2)*	*1 (0.1)*
Mother education			
Low	243 (31.4)	169 (31.4)	228 (32.9)
High	308 (39.7)	229 (42.5)	289 (41.8)
Don’t know	206 (26.6)	133 (24.7)	166 (24.0)
*Missing*	*18 (2.3)*	*8 (1.5)*	*9 (1.3)*
Father education			
Low	291 (37.5)	198 (36.7)	253 (37.0)
High	238 (30.7)	187 (34.7)	237 (34.2)
Don’t know	215 (27.7)	138 (25.6)	182 (27.0)
*Missing*	*31 (4.0)*	*16 (3.0)*	*17 (2.5)*
Chronic diseases, yes	198 (25.5)	135 (25.0)	205 (29.6)
*Missing*	*6 (0.8)*	*4 (0.7)*	*5 (0.7)*
Low social acceptance^a^	212 (27.4)	148 (27.5)	198 (28.6)
*Missing*	*27 (3.5)*	*15 (2.8)*	*11 (1.6)*
Psychological distress^b^	107 (13.8)	76 (14.1)	131 (18.9)
*Missing*	*24 (3.1)*	*9 (1.7)*	*17 (2.5)*

Values are number, n (%) if not otherwise stated. Main analyses = participants without persistent MSK pain at baseline, secondary analyses = all participants with and without persistent MSK pain at baseline. *BMI* body mass index, *MSK* musculoskeletal; ^a^Subscale from Self-perception profile for adolescents scale. Low social acceptance ≤3.0. ^b^Hopkins symptom check list-10 (1–4), psychological distress = ≥ 1.85

### Prevalence of persistent MSK pain

In the sample including participants with and without persistent MSK pain at baseline (*n* = 692), the prevalence of persistent MSK pain was 19.1% (*n* = 132) at baseline and 18.1% (*n* = 125) at the two-year follow-up. The prevalence of severe persistent MSK pain at baseline was 10.7% (*n* = 74) and 8.7% (*n* = 60) at follow-up. Forty-four percent of those with persistent MSK pain at baseline had persistent MSK pain at follow-up.

### Two-year incidence of persistent MSK pain

The two-year incidence of persistent MSK pain was 10.9% (*n* = 59), including 4.8% (*n* = 26) that reported severe persistent MSK pain.

### Association between low social acceptance at baseline and persistent MSK pain at follow-up

In univariate analysis, those who reported low social acceptance had 1.8 (95% CI [1.0–3.1]) higher odds for incidence persistent MSK pain compared to those with normal/high levels of social acceptance. When adjusted for sex and chronic diseases, the association remained statistically significant (OR = 1.8, 95% CI [1.0–3.2]) (Table 2). The association between low social acceptance and incident severe persistent MSK pain at follow-up had an OR of 1.2 (95% CI [0.5–2.9]) in crude and adjusted analyses, but were not statistically significant (Table 2).

**Table 2:** Associations between low social acceptance at baseline and persistent musculoskeletal pain at follow-up, main analysis

Exposure Outcome		Crude		Adjusted^**a**^	
	Cases/total	OR	95%CI	OR	95% CI
**Low social acceptance**					
Persistent MSK pain	57/524	1.8	1.0–3.1	1.8	1.0–3.2
**Low social acceptance**					
Severe Persistent MSK pain	26/506	1.2	0.5–2.8	1.2	0.5–2.9

Analyses of participants with no persistent MSK pain at baseline

*MSK* musculoskeletal, *Severe MSK pain* pain intensity ≥5 (1–10)

^a^ Adjusted for sex and chronic diseases

In the secondary analyses, including all participants regardless of pain status at baseline, we found statistically significant associations between low social acceptance among peers at baseline and persistent MSK pain at follow-up in crude (OR = 1.7, 95% CI [1.1–2.6]) and adjusted analyses (OR = 1.7, 95% CI [1.1–2.6]) (Table 3). No statistically significant associations between low social acceptance and severe persistent MSK pain at follow-up were revealed in crude (OR = 1.4, 95% CI [0.8–2.5]) nor adjusted analyses (OR = 1.4, 95% CI [0.8–2.5]) (Table 3).

**Table 3:** Associations between low social acceptance at baseline and persistent musculoskeletal pain at follow-up, secondary analysis

Exposure Outcome		Crude		Adjusted^a^	
	Cases/total	OR	95%CI	OR	95% CI
**Low social acceptance**					
Persistent MSK pain	124/681	1.7	1.1–2.6	1.7	1.1–2.6
**Low social acceptance**					
Persistent Severe MSK pain	59 /616	1.4	0.8–2.5	1.4	0.8–2.5

Analyses of participants with and without persistent MSK pain at baseline

*MSK* musculoskeletal, *Severe MSK pain* pain intensity ≥5 (1–10)

^a^Adjusted for sex and chronic diseases

### Moderation analyses

The moderation analysis revealed no effect modification of psychological distress on the relationship of low social acceptance among peers and persistent MSK pain 2 years later among adolescents without persistent MSK pain at baseline (*p* = 0.89). Univariate logistic regression analyses stratified by levels of psychological distress revealed similar associations among participants reporting a high level of psychological distress (OR = 1.8, 95% CI [0.2–10.6]) and participants with a low level of psychological distress (OR = 2.0, 95% CI [1.1–3.7]). No effect modification of psychological distress was found (*p* = 0.23) in the secondary analysis of all participants.

## Discussion

In this longitudinal study among Norwegian adolescents with no persistent MSK pain at baseline, low social acceptance among peers in the first year of upper secondary school was significantly associated with persistent MSK pain 2 years later, but not with severe persistent MSK pain. Sex and having chronic diseases did not confound these associations. Psychological distress did not modify the association between social acceptance among peers and persistent MSK pain. Secondary analysis, including participants with and without persistent MSK pain at baseline, also revealed that low social acceptance among peers was associated with persistent MSK pain 2 years later.

Our results indicating a significant association between low social acceptance among peers and persistent MSK pain is in line with two other studies investigating social relationships and persistent MSK pain in adolescents. Wurm et al. found a significant association between peer-related stress and persistent MSK pain in 13 to 15-year-old adolescents, and the association was mediated by worries in girls [13]. Loneliness was associated with spinal pain in a cross-sectional study of Danish adolescents [12]. To the best of our knowledge, the effect modification of psychological distress on the longitudinal relationship of low social acceptance among peers and persistent MSK pain has not been investigated in previous studies. However, a meta-analysis from 2020 reported that poor peer relationships and emotional well-being were closely linked [15], and a systematic review of children and adolescents found that adolescents with psychological distress have a higher longitudinal risk of MSK pain [6]. Nevertheless, psychological distress might have another important role in the relationship between social acceptance among peers and persistent MSK pain, such as a mediator or confounder, rather than an effect modifier.

Several explanations exist for the relationship between social relationships and pain. Adolescents perceiving themselves as having difficulties being liked among peers might experience this as “painful,” and one theory is that this “social pain” and physical pain affect the same brain areas as illustrated in functional MRI studies [30, 31]. Further, social pain and physical pain also share other common influential factors, such as cognition, behavioural and neurophysiological responses, and affective states [32]. Studies also indicate some shared neural substrates [33], indicating that people sensitive to physical pain are also sensitive to social pain. This is supported by experimental studies showing that people sensitive to experimental pain also self-report a higher sensitivity to social pain, and vice versa [34].

No statistically significant association was found between low social acceptance among peers and the secondary outcome, severe persistent MSK pain. Further, the point estimate was lower (OR = 1.2, 95% CI [0.5–2.9]) than in the main analyses (OR = 1.8, 95% CI [1.0–3.2]), suggesting a reduction in the strength of the association. However, in the secondary analysis of all study participants, the OR was 1.4 and the confidence interval ranging from 0.8–2.5 indicating a higher level of uncertainty in the direction and magnitude of the association. A potential lack of statistical power may explain the wide confidence interval crossing the non-significant cut-off since the secondary outcome had much fewer cases than the primary outcome (26 vs 57 cases).

### Implications

This study contributes to the knowledge regarding social factors and their potential impact on developing persistent MSK pain among adolescents. The study supports the current understanding that MSK pain often is a condition in which social factors play a role in the persistence of pain experience [35]. The findings also suggest that low social acceptance among peers could potentially be a meaningful target for future interventions among adolescents. Health professionals, teachers, and other professionals working with adolescents should be aware of the importance of peer relations to adolescents’ health. The effect modification of psychological distress should be further explored in larger studies, and the potential mechanisms of psychological distress could be investigated in mediation analyses. Studies of other possible mediating or interacting variables such as loneliness and coping are required to obtain a deeper understanding of the underlying mechanisms of the association between social acceptance and persistent MSK pain.

### Strengths and limitations

This study’s strength is the prospective design and the population-based sample from both urban and rural regions. The response rate at baseline was high, and the dropout was low. This study is the first to investigate the association of social acceptance among peers and persistent MSK pain in adolescents. A limitation of the study is the small sample size including few cases with MSK pain, which was a limiting factor especially for the moderation analysis. Power issues may explain why psychological distress did not modify the relationship between social acceptance and persistent MSK pain. Further, the cut-off classifying adolescents into high or low psychological distress levels is not validated explicitly for adolescents. Confounding factors were included based on previous empirical findings, but we were unable to adjust for socioeconomic status due to many adolescents not knowing their parent’s education level. More males than females were lost to follow-up, and more students lost to follow-up did not know their parent’s educational level. Another limitation is that no well-established cut-off value exists for the social acceptance scale, so our cut-off value was derived based on previous normative data [20]. This may have led to an unclear or arbitrary categorisation of adolescents into social acceptance levels. Further, the questionnaire used to measure persistent MSK was specifically developed for the Fit Futures study, and has not been validated to measure MSK pain in adolescents or in adults.

## Conclusions

Low social acceptance during the first year of upper secondary school was associated with persistent but not severe persistent MSK pain 2 years later. Psychological distress was not an effect modifier in these relationships. Our findings suggest that helping adolescents build healthy, accepting peer relations may prevent future persistent MSK pain.

## Supplementary Information

**Additional file 1.** STROBE Statement—checklist of items that should be included in reports of observational studies.

**Additional file 2.** Analyses of baseline characteristics of study participants and participants lost to follow-up.

## Data Availability

Data cannot be shared publicly because of restrictions from the Regional Committees for Medical and Health Research Ethics (post@helseforskning.etikkom.no) in accordance with Norwegian law, as participants in the Fit Future Study have not given consent to public sharing of their data. Therefore, these data are only available upon appropriate request to the Fit Future Study group at fitfutures@uit.no.

## Abbreviations

BMI: Body mass index
CI: Confidence interval
FF: Fit futures
MSK: Musculoskeletal
OR: Odds ratio
SD: Standard deviation
STROBE: Strengthening the Reporting of Observational Studies in Epidemiology statement
YLD: Years lived with disability
