# Supplementary material for: Low social acceptance among peers increases the risk of persistent musculoskeletal pain in adolescents. Prospective data from the Fit Futures Study
Source: BMC Musculoskelet Disord. 2022 Jan 13;23:44. doi: 10.1186/s12891-022-04995-6 (PMC8756715; doi:10.1186/s12891-022-04995-6)
Supplement: Supplementary file 2 — Additional file 2. Analyses of baseline characteristics of study participants and participants lost to follow-up. [file 12891_2022_4995_MOESM2_ESM.docx]

| **Variables** |  | **Study sample N=539** |  | **Lost to follow-up n= 219** |  | **p-value** |
| --- | --- | --- | --- | --- | --- | --- |
| Sex, females (%) |  | 280 (51.9%) |  | 71 (32.4) |  | <0.00* |
| Age |  | 16.1 (0.5) |  | 16.1 (0.6) |  | 0.42 |
| BMI  Thinness  Normal weight  Overweight(obese  *Missing* |  | 29 (5.4)  392 (72.2)  117 (21.7)  *1 (0.2)* |  | 8 (3.7)  149 (68.0)  60 (27.4)  *2 (0.9)* |  | 0.19 |
| Mother education  Low  High  Don’t know  *Missing* |  | 169 (31.4)  229 (42.5)  133 (24.7)  *8 (1.5)* |  | 69 (31.5)  71 (32.4)  69 (31.5)  *10 (4.6)* |  | 0.03* |
| Father education  Low  High  Don’t know  *Missing* |  | 198 (36.7)  187 (34.7)  138 (25.6)  *16 (3.0)* |  | 85 (38.8)  46 (21.0)  73 (33.3)  *15 (6.8)* |  | 0.00* |
| Chronic diseases, yes (%)  *Missing* |  | 135 (25.0)  *4 (0.7)* |  | 60 (27.4)  1 (0.5) |  | 0.47 |
| Low social acceptance^§^  *Missing* |  | 148 (27.5)  *15 (2.8)* |  | 59 (26.9)  *12 (5.5)* |  | 0.95 |
| Psychological distress^⸸^  *Missing* |  | 76 (14.1)  *9 (1.7)* |  | 29 (13.2)  *14 (6.4)* |  | 0.96 |
| BMI=body mass index; MSK=musculoskeletal;^§^Subscale from Self-perception profile for adolescents scale. Low social acceptance = ≥ 3.0.  ^⸸^Hopkins symptom check list-10 (1-4), psychological distress = ≥ 1.85 | | | | | | |

**Additional File 2. Analyses of baseline characteristics of study participants and participants lost to follow-up.**
